# Pile caps with inclined shear reinforcement and steel fibers

**DOI:** 10.1038/s41598-022-14416-2

**Published:** 2022-06-16

**Authors:** Aaron Nzambi, Lana Gomes, Cledinei Amanajás, Francisco Silva, Dênio Oliveira

**Affiliations:** 1 Department of Civil Engineering, Federal University of Pará, Belém, PA 66075-110 Brazil; 2 Department of Civil Engineering, University of Amazônia, Belém, PA 66065-205 Brazil

**Keywords:** Civil engineering, Structural materials

## Abstract

This experimental investigation presents results and a discussion on a series of four reinforced concrete pile caps with and without steel fibers, measuring 400 × 400 × 1000 mm^3^, which were tested under concentric loading. The study set the steel fiber and the inclined shear reinforcement as variables. The fiber volume fraction was 1.5%, and the concrete compressive strength was 25 MPa. The results showed a tendency to increase ductility and ultimate strength with the use of inclined shear reinforcement; the same behavior was observed with the addition of steel fibers, improving the performance of the tested pile caps. This study opens the possibility of designing slender pile caps, especially when associated with both analyzed parameters.

## Introduction

Foundations are among the most important structural elements used for load transfer from a building to piles or caissons. Pile caps can be classified as rigid or flexible based on criteria similar to those used for shallow foundations. Therefore, the larger and more rigid the structure is, the more complex its design becomes. According to NBR 6118^1^, rigid pile caps are not prone to diagonal tension failure; most often, failure occurs in compression struts^2^. However, some doubt remains as to whether this behavior can be modified with strut strengthening. In the literature, studies^3–5^ have suggested limiting the height of the block as a function of the angle of inclination of the strut, ranging from 45° to 55°; this may result in an overdesign of the structure. Fighting the shear cracks through reinforcement mechanisms that can improve the performance of the blocks is an ideal way to optimize the design and safety of such structural elements. This can be achieved by using shear reinforcement and/or steel fibers to improve the mechanical properties of concrete in terms of shear, compression, and flexure; in addition, steel fiber reinforced concrete (SFRC) enhances performance in energy absorption, cracking control, and ductility^6,7^. The use of SFRC is being expanded, and its applications range from floor pavements to special structural elements or those subjected to an aggressive or corrosive environment without affecting their durability^8^.

Thus, this experimental study explores the effectiveness of inclined reinforcement and the contribution of steel fibers in improving the performance of concrete pile caps subjected to shear forces since, economically, a considerable gain in strength increase can make this approach feasible and decrease the volume of concrete in pile caps.

## Calculation model

**Figure 1:**
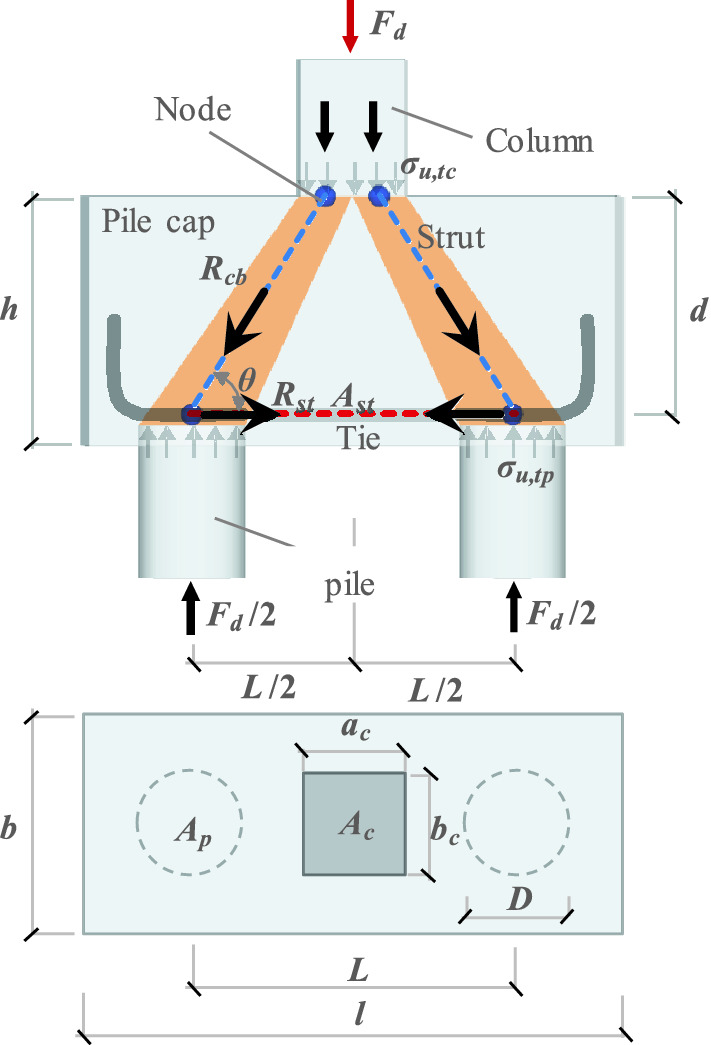
Strut-and-tie model for a two-pile cap (Note: *F*_*d*_/2 is the column load for a pile; *R*_*cb*_ is the compressive force on the strut; *R*_*st*_ is the tensile force on the tie; *θ* is the angle of inclination of the strut).

In the present study, the pile caps were designed using the strut-and-tie method (STM), which is the most widespread calculation model for the design of rigid pile caps. This design is based on the experimental works previously developed by Blévot and Frémy^4^. The model consists of designing a spatial truss inside a pile cap using tension and compression bars that are connected through nodes, as shown in Fig. 1.

The effective depth (*d*) of the pile cap is given by Eq. 1. To ensure adequate structural behavior of the pile cap, Blévot and Frémy^4^ recommended that *θ* should be within a 45° ≤ *θ* ≤ 55° range. For the minimum spacing between the piles adopted in the work, the distance *L* was equal to three times the edge distance of the pile (*D*), according to the recommendations of Moraes^9^, (*L* ≥ 3⋅*D*).

1 $$d = \tan {\uptheta } \cdot \left( {0.5 \cdot L - 0.25 \cdot {\text{a}}_{c} } \right)$$

where $L$ is the spacing between piles; ${\text{a}}_{c}$ is the larger side of the column.

The determinations of the acting stresses near the column and the pile are calculated through Eqs. (2) and (3), respectively. The equations also define the normative verification limits for the design.

2 $$\sigma_{u,tc} = \frac{{F_{d} }}{{A_{c} \cdot {\text{sin}}^{2} {\uptheta }}} \le 1.4 \cdot f_{c}$$

3 $$\sigma_{u,tp} = \frac{{F_{d} }}{{2 \cdot A_{p} \cdot {\text{sin}}^{2} {\uptheta }}} \le 0.85 \cdot f_{c}$$

where $\sigma_{u,tc}$ and $\sigma_{u,tp}$ represent the failure stresses of the strut in the regions of the column and the pile, respectively; *A*_*c*_ and *A*_*p*_ are the cross-sectional areas of the column and the pile, respectively; *F*_*d*_ is the design load; and fc is the concrete compressive strength.

The longitudinal reinforcement area of the tie over the piles was calculated according to Eq. (4). Equation (5), proposed by Delalibera and Giongo^10^, was used to calculate the tensile force in the tie.

4 $$A_{st} = \frac{{R_{st} }}{{f_{ys} }}$$

5 $$R_{st} = \frac{{F_{d} \cdot \left( {2L - a_{c} } \right)}}{8 \cdot d}$$

where *A*_*st*_ is the total area of longitudinal steel reinforcement in the tie, *R*_*st*_ is the tensile force on the tie, and *f*_*ys*_ is the yield strength of the longitudinal steel reinforcement.

## Experimental setup

### Materials and methods

**Figure 2:**
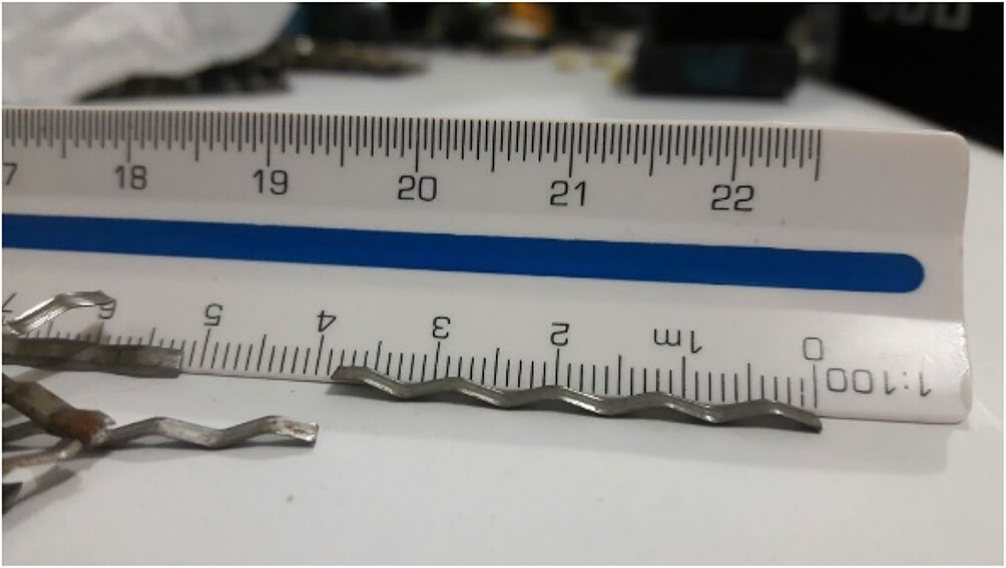
Flat crimped steel fiber. (Reprinted with permission from Nzambi et al.^14^.

Cement, coarse aggregate, fine aggregate, and water–cement ratio (*w*/c) were mixed in a 1:2.90:2.10:0.55 proportion. Superplasticizer additive was used to maintain the constant workability of concrete. Table 1 lists the constituents used for the mixtures, and Table 2 lists the mechanical properties of the crimped steel fiber. Flat crimped type C fiber (Fig. 2), classified according to NBR 15530^11^, was used in this research because, according to Soroushian and Bayasi^12^ and Mahakavi and Chithra^13^, hooked-end and crimped fibers are more effective at improving the performance of structural elements as a result of their additional anchoring mechanisms^14^. The concrete test specimens were molded and cured for 28 days in the laboratory with 85% relative air humidity. Table 3 presents the results of the characterization tests at 7, 14, and 28 days. There was no significant difference between the plain concrete and the concrete with fiber, so average values were adopted for both mixtures: 25.8 MPa, 1.9 MPa, and 28.4 GPa, respectively, for compressive strength (*f*_*c*_), tensile strength (*f*_*ct*_), and modulus of elasticity (*E*_*c*_).

**Table 1:** Composition of concrete.

Constituents	Type	*ρ* (kg/m^3^)	Weight per unit volume (kg)	
			Mixture 1	Mixture 2
Cement	Portland CPII-Z-32RS	3100	269.52	269.52
Small aggregate	Sand (avg. 2.7 mm)	2830	781.62	781.62
Large aggregate	Coarse aggregates (dmax = 10 mm)	2600	566.00	566.00
Fiber	Crimped steel fiber	7850	–	85.84*
Admixture	Superplasticizer	–	–	–
Water	pH < 9	1000	148.24	148.24

w/c = 0.55 for all mixtures and slump test workability 140 mm; *V_*f*_ = 1.5% = 117.75 kg/m^3^, *C*_*f*_ = *V*_*c*_ × *V*_*f*_ = 0.729 m^3^ × 117.75 kg/m^3^ = 85.84 kg; ***ρ*** is the density; *C*_*f*_ is the fiber consumption; *V*_*c*_ is the volumetric fraction of the concrete; and *V*_*f*_ is the volumetric fraction of the fiber.

**Table 2:** Mechanical properties of crimped steel fibers.

*d*_*f*_ (mm)	*l*_*f*_ (mm)	*l*_*f*_*/d*_*f*_	*f*_*u,f*_ (MPa)	*E*_*f*_ (GPa)
~ 1.0	38.0	38.00	900.00	200.00

*d*_*f*_ = equivalent diameter of fiber; *l*_*f*_ = fiber length; *l*_*f*_/*d*_*f*_ = aspect ratio; *f*_*u,f*_ = ultimate tensile strength of fiber; and *E*_*f*_ = elastic modulus of fiber.

**Table 3:** Results for characterization of concrete (avg. values).

Age of concrete (Days)	Number of cylindrical specimens	*f*_*c,m*_ (MPa)	*f*_*ct,m*_ (MPa)	*E*_*c*_ (GPa)
07	3	14.30	1.24	21.18
14		16.90	1.38	23.02
28		25.80	1.90	28.44

The steel bars used in the tests were classified according to NBR 7480^15^. Their mechanical properties were determined through axial tensile tests, following the recommendations of NBR ISO 6892-1^16^. Three samples were used in the tensile test; the test bars measured 5.0 mm, 10.0 mm and 12.5 mm in diameter and were used in the stirrups, inclined shear reinforcement and flexural reinforcement, respectively. Table 4 presents the mechanical properties of the steel used.

**Table 4:** Mechanical properties of the reinforcements used.

∅_s_ (mm)	*ε*_*ys*_ (‰)	*f*_*ys*_ (MPa)	*f*_*us*_ (MPa)	*E*_*s*_ (GPa)	Location
5.0	4.63	529.50	550.00	201.70	Stirrups
10.0	2.42	500.00	556.00	206.61	Inclined shear reinforcement
12.5	3.05	610.30	716.00	200.00	longitudinal reinforcement

∅_s_ = steel bar diameter; *ε*_*ys*_ = strains; *f*_*ys*_ = yield stresses; *f*_*us*_ = ultimate tensile strength of steel bar; *E*_*s*_ = modulus of elasticity.

### Characteristics of the samples

**Figure 3:**
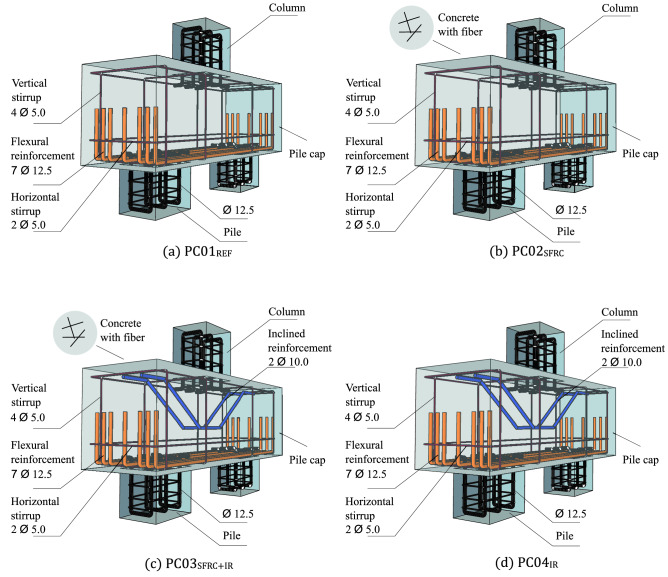
Details of the two-pile caps.

**Figure 4:**
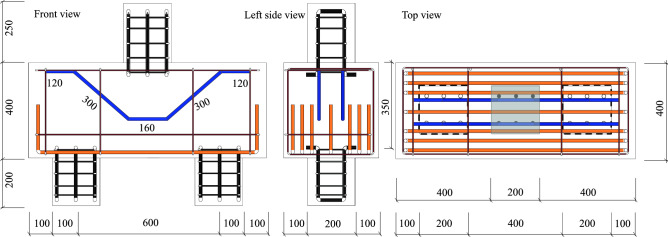
Pile cap dimensions (in mm).

The design of the pile caps followed the strut-and-tie method presented in “Calculation model” section^4^. The dimensions were constant for all pile caps, as shown in Table 5. The column located on the upper surface of the piles had a cross-section of 200 × 200 mm^2^ with a height of 250 mm. The piles had the same dimensions for the cross section, but their height was 200 mm (Figs. 3, 4). The compressive strength of the concrete was 25.8 MPa for all pile caps mixtures. The longitudinal reinforcement and horizontal and vertical stirrups were the same. Pile caps PC03_SFRC+IR_ and PC04_IR_ were made with two inclined bars with a diameter of 10.0 mm. The spacing between the bars was 100 mm, and each bar had a length of 1000 mm. The following are the other characteristics of each pile cap:

- PC01_REF_: concrete cast without the addition of steel fiber and inclined shear reinforcement, as shown in Fig. 3a;
- PC02_SFRC_: concrete cast with the addition of steel fiber (*V*_*f*_ = 1.5%) but without inclined shear reinforcement, as shown in Fig. 3b;
- PC03_SFRC+IR_: concrete characteristic equal to PC02_SRC_ + inclined shear reinforcement, as shown in Fig. 3c;
- PC04_IR_: concrete characteristic equal to PC01_REF_ but with inclined shear reinforcement, as shown in Fig. 3d.

**Table 5:** General characteristics of the pile caps.

Pile cap	*a*_*c*_ (mm)	*d* (mm)	*b* (mm)	*h* (mm)	*l* (mm)	*L*(mm)	*V*_*f*_ (%)	Inclined shear reinforcement
PC01_REF_	200	350	400	400	1000	600	–	–
PC02_SFRC_							1.5	–
PC03_SFRC+IR_								Yes
PC04_IR_							–	

NOTE: REF = reference (plain concrete); SFRC = steel fiber reinforcement concrete; and IR = inclined reinforcement.

### Instrumentation

**Figure 5:**
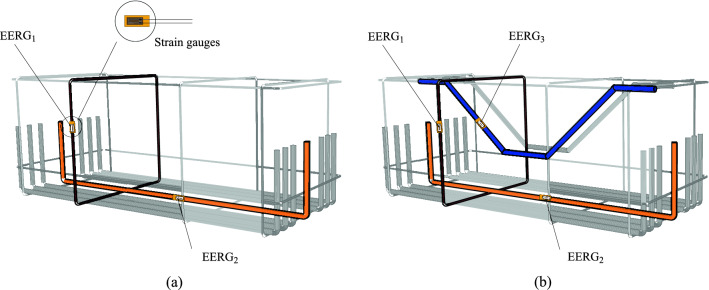
Position of strain gauges on the reinforcements: (**a**) for PC01_REF_ and PC04_IR_ and (**b**) for PC02_SFRC and_ PC03_SFRC+IR._

**Figure 6:**
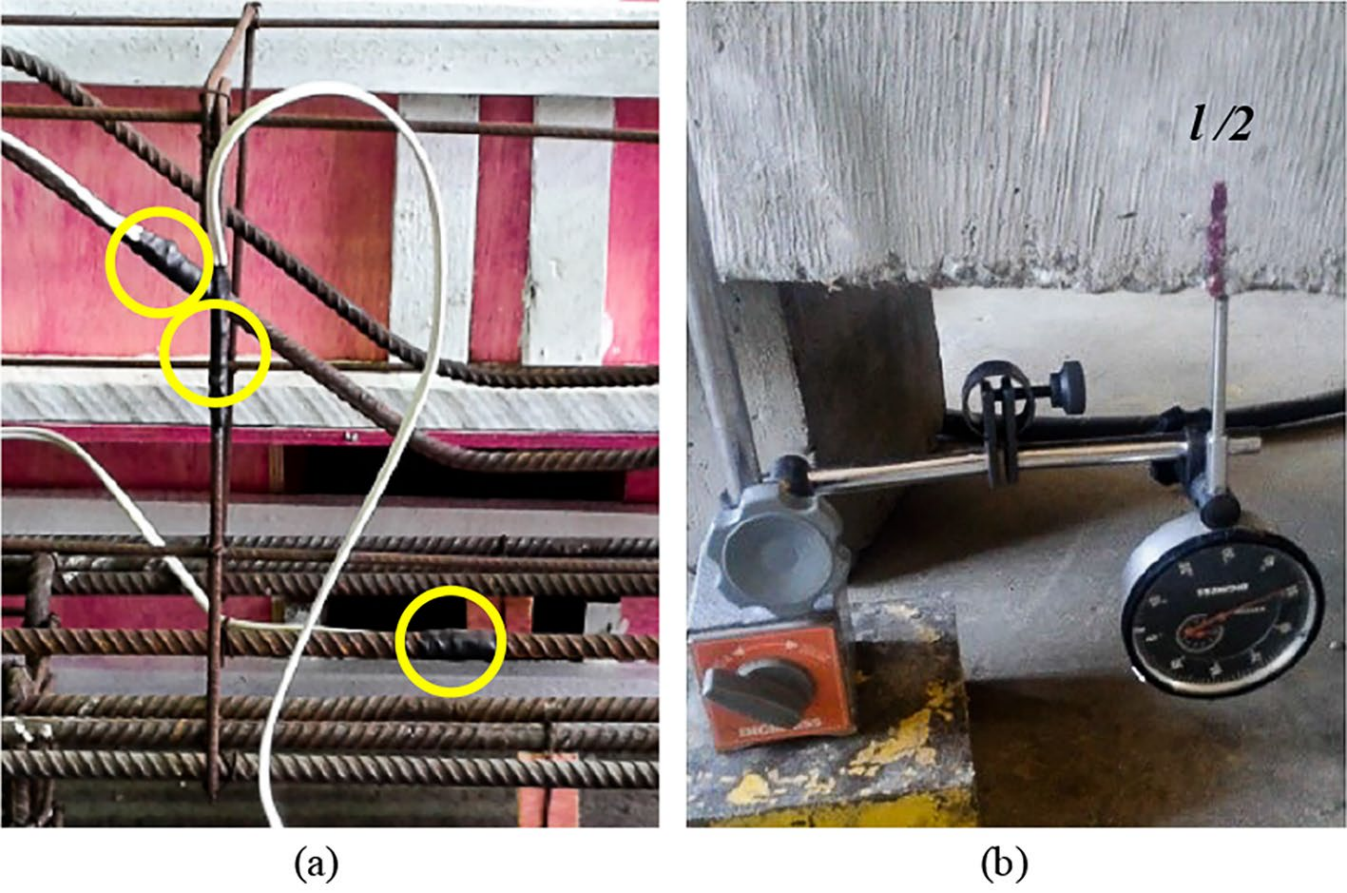
Instrumentation: (**a**) installed strain gauges and (**b**) deflectometer placement.

**Figure 7:**
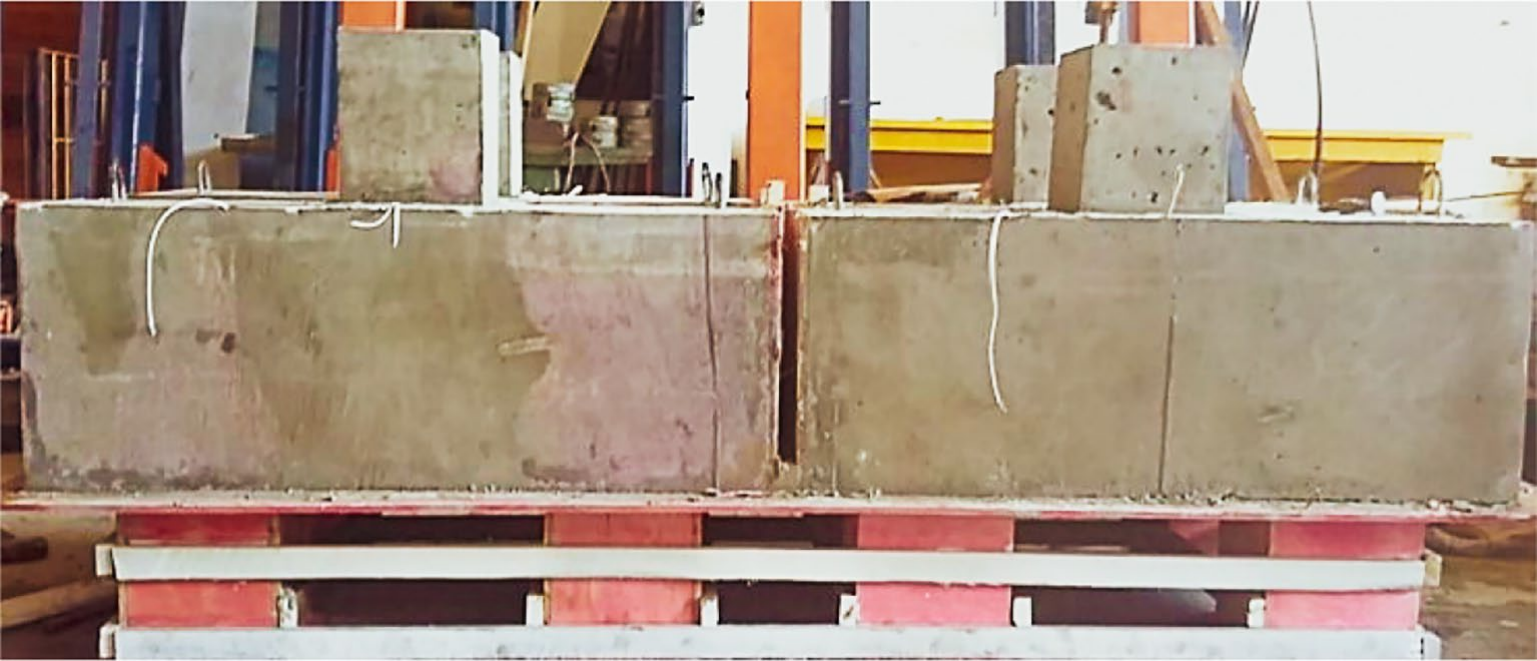
Pile cap specimens.

The strains in the steels were measured using electrical resistance strain gauges (EESG, *EXCEL Sensors*, PA-06-125AA-120L), positioned as shown in Fig. 5. Therefore, one strain gauge was installed in the middle of the central flexural reinforcement bar, one in the inclined shear reinforcement bar, and one in the vertical stirrup at the level of the point of intersection with the inclined reinforcement (Fig. 6a), to be in the same stress line caused by the compression strut. The vertical displacements were measured using a deflectometer placed at the bottom of the pile caps at a distance of *l*/2 = 500 mm, as shown in Fig. 6b. Figure 7 shows the final aspect of the pile caps ready for testing.

### Test setup

**Figure 8:**
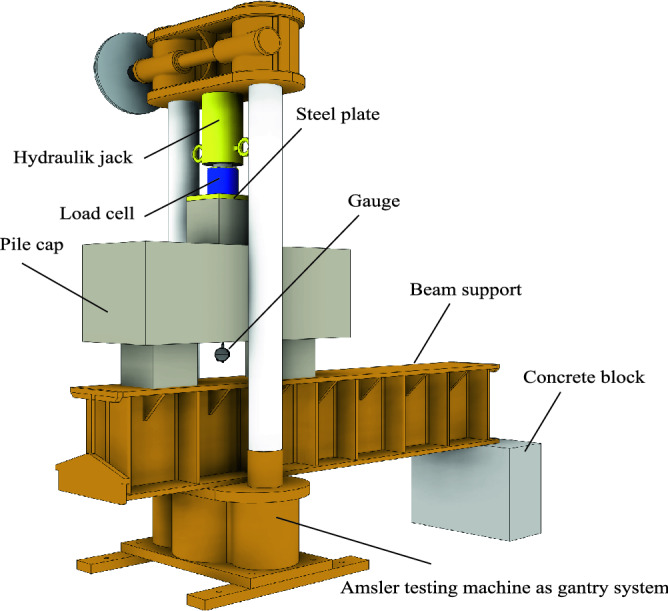
Test setup.

All pile caps were subjected to centered loading and applied on the face of the column. The test setup was composed of a hollow hydraulic jack and a hydraulic pump (Enerpac). Both the jack and the pump had a 1000 kN capacity, a digital load cell for 1000 kN, a precision of 500 N and an Amsler testing machine used as the gantry system. Figure 8 shows the test system. In all test specimens, an initial load was applied to eliminate looseness.

## Results and discussion

Table 6 presents the results of the experimental bearing capacity. In terms of strength gain, PC02_SFRC_ and PC04_IR_ presented an increase in the ultimate strength of approximately 40% compared to block PC01_REF_. Furthermore, the fibers increased the load-bearing capacity of the PC03_SFRC+IR_ pile cap by 7% when used with inclined reinforcement compared to PC04_IR_.

**Table 6:** Ultimate failure loads observed during testing.

Pile cap	*Strut angle, θ* (°)	*Experimental*	
		*Ultimate failure Load, V*_*u*_ (kN)	Strength increase, *V*_*u,PileCaps*_*/V*_*u,REF*_ (%)
PC01_REF_	54.46*	489.00	–
PC02_SFRC_		680.00	39
PC03_SFRC+IR_		720.00	47
PC04_IR_		685.00	40

*V*_*u,Pilecaps*_ = failure loads of the pile caps with steel fibers and/or inclined reinforcement *V*_*u,REF*_ = failure loads of the pile caps without steel fibers and/or inclined reinforcement. ^*^From Eq. (1), $\tan \theta = d/\left( {0.5 \cdot L - 0.25 \cdot a_{c} } \right) = 350/\left( {0.25 \cdot 600 - 0.25 \cdot 200} \right) = 1.4 \to arctn\theta = 54.46$.

### Load–displacement ratio and tenacity

**Figure 9:**
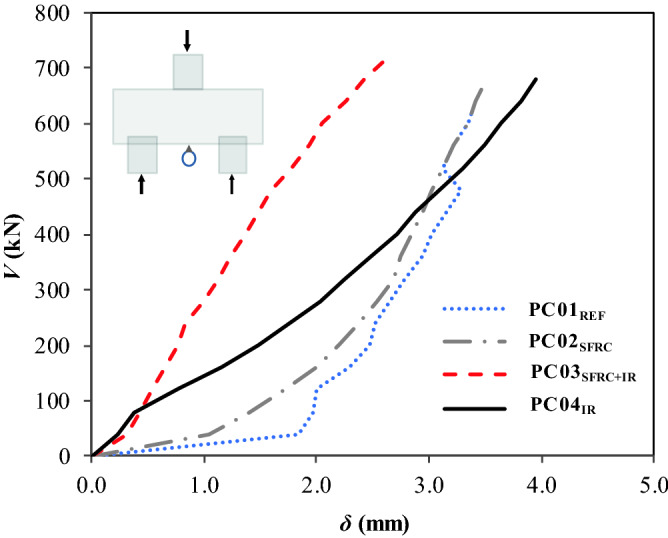
Load–displacement ratio*.*

This study focused only on the displacement in the center of the pile caps (*l*/2 = 500 mm); this way, the analyses were made based on the load–displacement (*V *− *δ*) ratio and the quantities that characterize this ratio. Although PC02_SFRC_ and PC04_IR_ had almost the same ultimate strength, Table 7 and Fig. 9 show a slight decrease in displacements with the addition of steel fiber; this effect was more pronounced with PC03_SFRC+IR_, which showed lower displacements, at a 20%, and 44% reduction, respectively, compared to PC01_REF_ and PC04_IR_.

**Table 7:** Parameters defining the *V* − *δ* ratio.

Pile cap	*V*_*u*_ (kN)	*δ*_*u*_ (mm)	*T*_*E*_ (kJ)	*δ*_*u,Pilecaps*_*/δ*_*u,REF*_	*T*_*E,Pilecaps*_*/T*_*E,REF*_
PC01_REF_	489.00	3.28	3.62	–	–
PC02_SFRC_	680.00	3.47	7.02	1.06	1.94
PC03_SFRC+IR_	720.00	2.61	10.02	0.80	2.77
PC04_IR_	685.00	3.93	12.14	1.20	3.35

An analysis was made of tenacity (*T*_*E*_), that is, the capacity of the pile caps to absorb strain energy (Table 7), based on the *T*_*E,Pilecaps*_*/T*_*E,REF*_ ratio. Clearly, the fibers and the inclined reinforcement helped to improve the tenacity of the pile caps; however, the most relevant values in terms of *T*_*E*_ were found for PC04_IR_, with *T*_*E,Pilecaps*_*/T*_*E,REF*_ = 3.35, where *T*_*E,Pilecaps*_ and *T*_*E,REF*_ were the tenacity of the pile caps with some type of additional reinforcement and the reference tenacity, respectively. In terms of displacement, the gains of the pile caps with SFRC and inclined shear reinforcement were not so substantial, mainly for PC03_SFRC+IR_ (with hybrid reinforcement). It is assumed that the steel fibers inhibited the action of the inclined shear reinforcement, and for this reason, the results of that pile cap were below expectations in terms of ductility. However, this structural element clearly had major strength gains. Finally, it should be noted that for PC04_IR,_ the *δ*_*u,Pilecaps*_*/δ*_*u,REF*_ ratio = 1.20 was more significant.

### Mobilization of the flexural reinforcement

**Figure 10:**
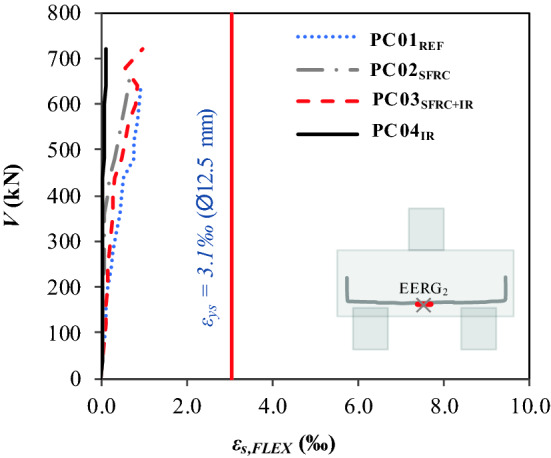
Load–strain ratio of the flexural reinforcement.

Table 8 shows the maximum strains of the flexural reinforcement *ε*_*fu*_, as expressed in the load–strain ratio. This information enables the analysis of the *ε*_*fu,Pilecaps*_*/ε*_*fu,REF*_ ratio and the angular constant *k*. The angular constant, determined by *k* = *V/ε*_*f*_, evaluates the tangent of the slope angle of the linear portion of the *V * − *ε*_*f*_ ratio. The *ε*_*fu,Pilecaps*_*/ε*_*fu,REF*_ ratio shows that the hybrid use of SFRC with inclined reinforcement (PC03_SFRC+IR_) had the best performance compared to PC01_REF_; that is, the increase in fiber consumption strengthened the reinforcement under tensile stress since the pile caps with SFRC had *ε*_*fu,Pilecaps*_*/ε*_*fu,REF*_ ∈ [0.9–1.3]. Moreover, in general, there was no yield stress in the reinforcements monitored in the pile caps, with *ε*_*fu*_ < *ε*_*sy*_ = 3.05 ‰ (Fig. 10). The evaluation of the angular constant shows that the responses in the elastic phase are within normal limits for the values presented, at *k* ∈ [4000–10000].

**Table 8:** Parameters that define the *V* − *ε*_*f*_ ratio.

Pile cap	*V*_*u*_ (kN)	*ε*_*fu*_ (‰)	*k*	*ε*_*fu,pilecaps*_*/ε*_*fu,REF*_	Failure mode
PC01_REF_	489.00	0.74	4090.9	–	Shear
PC02_SFRC_	680.00	0.68	6153.9	0.92	
PC03_SFRC+IR_	720.00	0.95	4500.0	1.28	
PC04_IR_	685.00	0.10	10,000.0	0.14

### Mobilization of the inclined shear reinforcement

**Figure 11:**
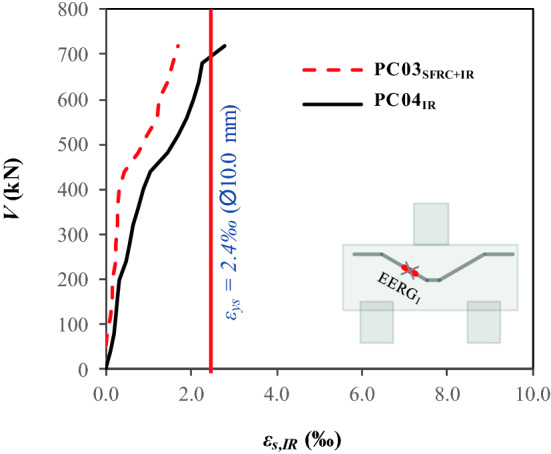
Load–strain ratio of the inclined reinforcements.

On this occasion, only pile caps PC03_SFRC+IR_ and PC04_IR_ were analyzed, as they have inclined shear reinforcement. In Fig. 11, the results show that strain in the instrumented reinforcement of PC04_IR_ was higher than yield strain, with *ε*_*su, IR*_ > *ε*_*sy*_ = 2.42‰. However, for the pile cap with hybrid composition (fibers + inclined reinforcement), the inclined reinforcement did not have yield stress. This finding suggests that the steel fibers assumed the function of reinforcement and inhibited the strain of the inclined reinforcement. In summary, the results indicate that the use of inclined shear reinforcement together with steel fibers can lead to oversizing, i.e., the fiber restricts the action of the reinforcement and vice versa. In addition, the pile caps had similar gains in strength, ductility and tenacity, with the exception of the reference pile cap; that is, it is hardly practical and economical to use steel fibers together with inclined shear reinforcement.

### Mobilization of the stirrups

**Figure 12:**
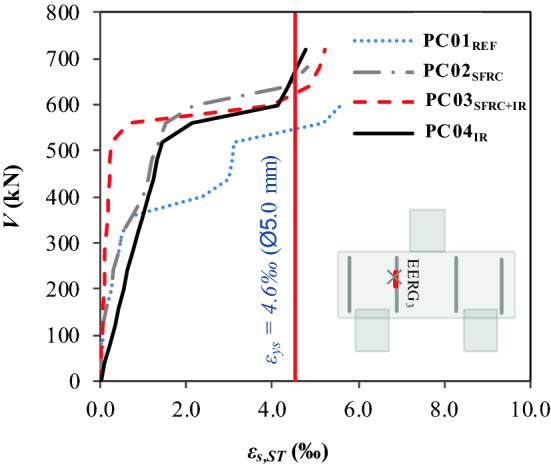
Load–strain ratio of the stirrups.

The analysis of stirrup mobilization was based on the results shown in the load-strain graph, *V* − *ε* (Fig. 12). The graph shows that, in general, the results had different bilinear behaviors, since one section represents the elastic phase (without the appearance of diagonal cracks), while the second section corresponds to the beginning and progress of shear cracks.

Table 9 shows the coordinates that define the ultimate load (*V*_*u*_ − *ε*_*su*_) as well as the coordinate indicating the start of the second linear section (*V*_*2L*_ − *ε*_*2L*_). The result for strain *ε*_*su*_ shows that yield stress occurred in the transverse reinforcement of all pile caps, *ε*_*su*_ > *ε*_*sy*_ = 4.63 ‰. The *ε*_*su,Pilecaps*_*/ε*_*su,REF*_ ratio clearly shows that the level of strain in the stirrups of the pile caps with additional reinforcement (steel fibers and/or inclined reinforcement) is lower than the level of the reference pile cap, namely, *ε*_*su,Pilecaps*_*/ε*_*su,REF*_ ∈ [0.80–0.95].

**Table 9:** Characterization of the *V* − *ε*_*s*_ ratio.

Pile cap	*V*_*u*_ (kN)	*ε*_*su*_ (‰)	*V*_*2L*_ (kN)	*ε*_*s2L*_ (‰)	*ε*_*su,Pilecaps*_/*ε*_*su,REF*_	*V*_*2L,Pilecaps*_/*V*_*2L,REF*_	*ε*_*s2L,Pilecaps*_/*ε*_*s2L,REF*_
PC01_REF_	489.00	5.65	320.00	0.51	–	–	–
PC02_SFRC_	680.00	4.85	320.00	0.52	0.86	1.00	1.02
PC03_SFRC+IR_	720.00	5.25	520.00	0.24	0.93	1.63	0.47
PC04_IR_	685.00	4.75	510.00	1.50	0.84	1.59	2.94

It can be seen that the reinforcement mechanism provided by the steel fibers and the inclined reinforcement reduces the stress in the reinforcement; therefore, for this study, the transverse reinforcement ratio or even the effective depth of the pile cap can be reduced. The analyses also show that the *V*_*2L,Pilecaps*_*/V*_*2L,REF*_ ∈ ratio [1.00–1.65] indicates that, at the beginning of transverse reinforcement mobilization, the stirrups of pile caps PC02_SRFC_, PC03_SRFC+IR_ and PC04_IR_ underwent more stress than those of the reference pile cap. However, with increased loading, the fibers and the inclined reinforcement tend to assume the function of the main reinforcement. In addition, with the imminence of failure, *ε*_*su,Pilecaps*_*/ε*_*su,REF*_ ∈ [0.80–0.95], which confirms the decrease in stress in the stirrups of the pile caps with additional reinforcements (steel fiber and/or inclined reinforcement).

### Failure of pile caps

**Figure 13:**
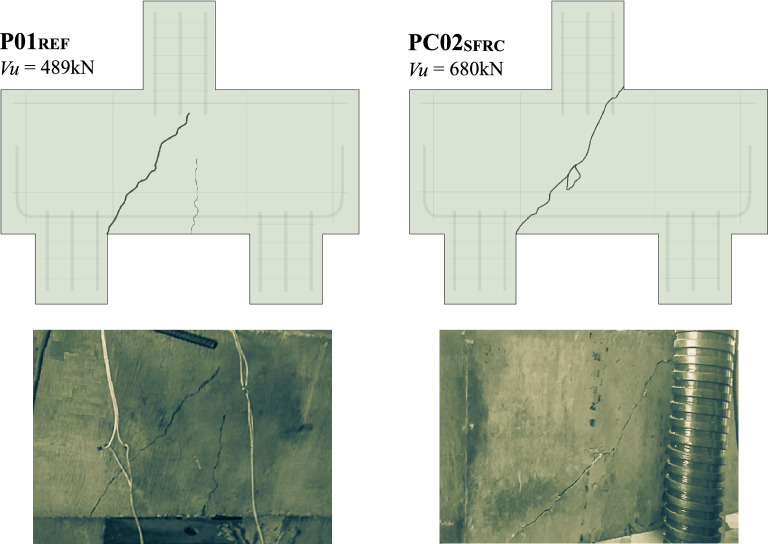
Final aspect of the pile caps after their failures without inclined shear reinforcement.

**Figure 14:**
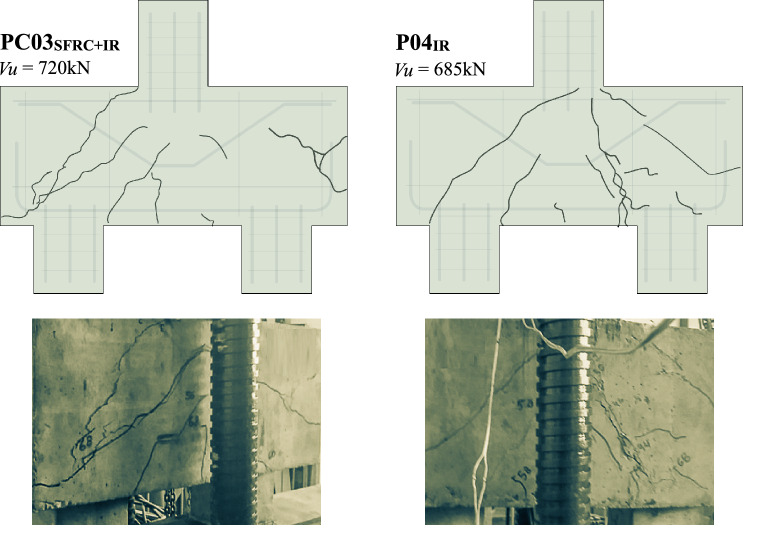
Final aspect of the pile caps after their failures with inclined shear reinforcement.

The analysis aims to register the integrity of the pile caps after the failure of these structural elements. Figures 13 and 14 reveal the fragility of these elements and their failure mode, namely, shear by diagonal strain. P01_REF_ was the weakest of all, with the least pronounced diagonal crack. The pile caps PC03_SFRC+IR_ and P04_IR_ demonstrated similar behavior, presenting the formation of multiple cracks. However, comparing PC03_SFRC+IR_ and PC02_SFRC_, it can be seen that the combination of inclined reinforcement and steel fibers enhances the bearing capacity, delaying the progression of cracks and inhibiting shear efforts, with the solicitation of the fibers in tension, as the load–strain and load–displacement graphs reveal. Therefore, steel fibers and shear reinforcement improved the ductility of the pile caps.

## Conclusions

The present experimental study has assessed steel fiber and inclined shear reinforcement performance in pile caps, whose variables were the use of steel fibers with and without inclined shear reinforcement. Based on the results, the following conclusions were observed:

- The use of the fibers provided the same value of the bearing capacity of the pile cap with the inclined reinforcement, with a strength gain of approximately 40%, comparing PC02_SFRC_ and PC04_IR_ with PC01_REF_.
- The fibers contributed to a 7% strength gain when the usage is combined with inclined shear reinforcement; comparing PC03_SFRC+IR_ with PC04_IR_.
- The analysis of the load–displacement ratio (*V-δ*) shows that the load-bearing capacity of the pile cap with steel fibers and inclined reinforcement (PC03_SFRC + IR_) had the best lower displacements, 20%, and 44% reduction, respectively, compared to PC01_REF_ and PC04_I_, and exhibited greater strain capacity, with *ε*_*fu*_/*ε*_*f*_ = 1.28.
- Additionally, the *V* − *δ* parameters enabled the assessment of not only strength but also the influence of steel fibers and inclined shear reinforcement on the ductility and tenacity of the pile caps. These properties increased significantly in pile caps with steel fibers and/or inclined shear reinforcement, especially PC04_IR_, with *δ*_*u,Pilecaps*_*/δ*_*u,REF*_ = 1.20 and *T*_*E,Pilecaps*_*/T*_*E,REF*_ = 3.35.
- The load–strain graph (*V* − *ε*_*s, IR*_) shows that the inclined shear reinforcement of PC03_SFRC+IR_ with hybrid composition (steel fibers + inclined shear reinforcement) did not carry yield stress.
- The mobilization of the stirrups shows that the levels of strain in the transverse reinforcements of pile caps PC02SFRC, PC03SFRC + IR, and PC04IR were lower than that of the reference pile cap. For this reason, the additional strengthening mechanisms reduced the shear efforts.
- The steel fibers restricted the strain of the inclined shear reinforcement. Therefore, it can be hypothesized that the strengthening mechanisms, under the conditions presented in this study, work satisfactorily by themselves, as the fibers tend to inhibit the action of the inclined shear reinforcement and vice versa.

## Data Availability

The datasets used and/or analyzed during the current study are available from the corresponding author on reasonable request.
