# Author Correction: Pile caps with inclined shear reinforcement and steel fibers

**DOI:** 10.1038/s41598-022-17919-0

**Published:** 2022-08-08

**Authors:** Aaron Nzambi, Lana Gomes, Cledinei Amanajás, Francisco Silva, Dênio Oliveira

**Affiliations:** 1 Department of Civil Engineering, Federal University of Pará, Belém, PA 66075-110 Brazil; 2 Department of Civil Engineering, University of Amazônia, Belém, PA 66065-205 Brazil

Correction to: *Scientific Reports* https://doi.org/10.1038/s41598-022-14416-2, published online 16 June 2022

The original version of this Article contained an error in the Materials and methods section, under the subheading ‘Characteristics of the samples’.

“PC04_IR_: concrete characteristic equal to PC01_REF_ but without inclined shear reinforcement, as shown in Fig. 3d.”

now reads:

“PC04_IR_: concrete characteristic equal to PC01_REF_ but with inclined shear reinforcement, as shown in Fig. 3d.”

The original Article has been corrected.

